# The Role of MRI in Groin Pain Syndrome in Athletes

**DOI:** 10.3390/diagnostics14080814

**Published:** 2024-04-14

**Authors:** Gian Nicola Bisciotti, Francesco Di Pietto, Giovanni Rusconi, Andrea Bisciotti, Alessio Auci, Marcello Zappia, Stefania Romano

**Affiliations:** 1 Kinemove Rehabilitation Centers, 54027 Pontremoli, Italy; bisciottign@yahoo.com; 2 Dipartimento di Diagnostica per Immagini, Pineta Grande Hospital, 81030 Castel Volturno, Italy; 3 Humanitas Research Hospital, 20089 Milan, Italy; bisciotti@libero.it; 4 Dipartimento delle Diagnostiche, Azienda USL Toscana Nord Ovest, 56121 Massa, Italy; alessio.auci@uslnordovest.toscana.it; 5 Department of Medicine and Health Science V. Tiberio, Università degli Studi del Molise, 86100 Campobasso, Italy; marcello.zappia@unimol.it; 6 Department of Radiology, S. Maria delle Grazie Hospital, 80078 Pozzuoli, Italy; stefromano@libero.it

**Keywords:** groin pain syndrome, MRI, inguinal hernia, prepubic aponeurotic complex, pubic osteopathy

## Abstract

Groin pain syndrome (GPS) is one of the most frequent injuries in competitive sports. Stresses generated in the lower limbs by quick turns and accelerations, such as in soccer, basketball or hockey, can produce localized regions of increased forces, resulting in anatomical lesions. The differential diagnoses are numerous and comprise articular, extra-articular, muscular, tendinous and visceral clinical conditions and a correct diagnosis is crucial if treatment is to be efficient. MRI is the gold standard of diagnostic techniques, especially when an alternative pathology needs to be excluded and/or other imaging techniques such as ultrasound or radiography do not lead to a diagnosis. This paper, based on the current literature, gives a comprehensive review of the anatomy of the pubic region and of the typical MRI findings in those affected by GPS. Many clinical conditions causing GPS can be investigated by MRI within appropriate protocols. However, MRI shows limits in reliability in the investigation of inguinal and femoral hernias and therefore is not the imaging technique of choice for studying these clinical conditions.

## 1. Introduction

In agreement with the definition approved during the Groin Pain Syndrome Italian Consensus Conference on terminology, clinical evaluation and imaging assessment in groin pain in athletes [1], groin pain syndrome (GPS) can be defined as follows:

“Any clinical symptom located in the inguinal-pubic-adductor area which affects sports activities and/or interferes with activities of daily living and requiring medical attention.”

GPS is an increasing problem in many sports that involve sudden changes in direction or kicking such as soccer, football, ice hockey, handball and rugby [2]. Indeed, due to numerous risk factors such as high activity loads and short recovery periods between matches, GPS is on the increase in several sports such as soccer, where it already represents 10–18% of all time-loss injuries [3]. Recent studies, based on time loss, report GPS incidences in soccer up to 2.1/1000 h of total exposure [4]. In such studies, injuries are recorded only if a player is unable to participate in soccer training and/or in a competition. [5,6,7,8,9]. There is evidence that the time-loss definition detects only one-third of all GPS injuries in male soccer players [10]. Therefore, the time-loss injury approach may be inappropriate for assessing GPS epidemiology, as the recorded data may represent only the tip of the iceberg of a greater more widespread problem [10]. Indeed, it is not uncommon for soccer players to continue training despite pain caused by GPS; thus, an overuse definition may be more appropriate for detecting certain types of GPS injury [11,12].

Three main categories of GPS are recognized, based both on the pathogenesis and the onset of symptoms [3,4], as follows:

- GPS from traumatic origin: the onset of pain follows an acute trauma, documented by medical history, clinical examination and imaging;
- GPS from functional overload: the onset may be insidious and unaccompanied by acute trauma or it may be attributed to a known cause;
- Long-standing GPS (LSGPS) or chronic GPS: the patient experiences a cohort of symptoms over a period of more than 12 weeks that does not respond to conservative therapy.

Numerous articular, extra-articular, muscular, tendinous and visceral clinical conditions may cause GPS. Given that MRI is particularly well suited for imaging soft tissues such as tendons, muscles and joints, including the symphysis and hip, it follows that this particular imaging technique plays a key role in the diagnosis of GPS [13,14,15].

It is important to remember that a single imaging examination technique does not exist for exhaustively studying the pubis [2]. Indeed, each diagnostic method (ultrasonography, conventional radiology, CT and MRI) presents its own individual limits when studying the various anatomical components of the pelvis [2,13,14] and yet, each method, in its own right, is important for diagnostic purposes [2,13,14]. However, MRI is the imaging method that not only has sufficient sensitivity to evaluate bone stress and joint injuries but also possesses the specificity for honing in on musculotendinous lesions and ruptures of the hip acetabular labrum when used with contrast mediums (i.e., MR arthrography) [2,13,14]. Since an optimal study of the anatomy of the pelvis is an essential diagnostic element in GPS, MRI must be recognized as one of the main diagnostic methods contributing to this area of diagnosis [13,14,15].

The aim of this article is to present the main musculoskeletal anomalies that can cause GPS and that can be investigated with MRI.

In order to better understand the causes of GPS, a description of the musculoskeletal anatomy of the groin region is essential.

## 2. Anatomy and Biomechanics of the Pubic Symphisis

**Figure 1:**
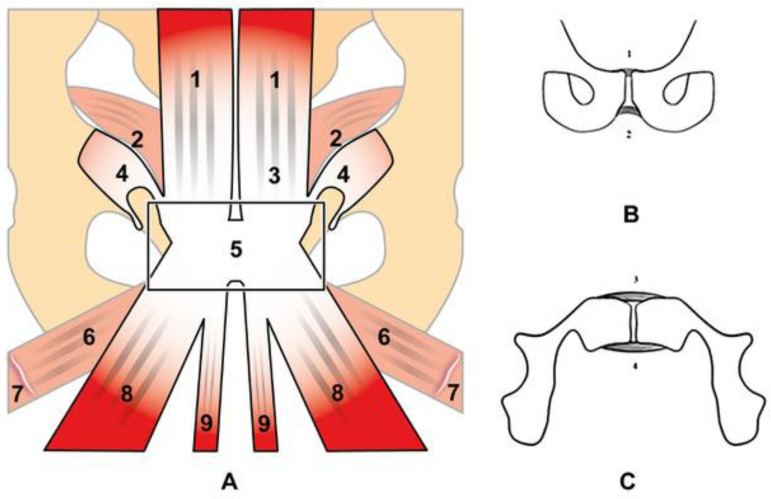
Schematic view of the tendon structure forming the prepubic aponeurotic complex (**A**) and schematic view of the pubic ligaments in coronal view (**B**) and axial view (**C**). The prepubic aponeurotic complex is formed by the anterior, inferior and superior pubic ligaments. Legends (**A**): (1) rectus abdominis; (2) transversus abdominis and internal oblique; (3) pyramidalis; (4) external oblique; (5) pre-pubic aponeurotic complex; (6) pectineus; (7) adductor brevis; (8) adductor longus; (9) gracilis. Legends (**B**,**C**): (1) superior pubic ligaments; (2) inferior pubic ligament; (3) anterior pubic ligament; (4) posterior pubic ligament.

**Figure 2:**
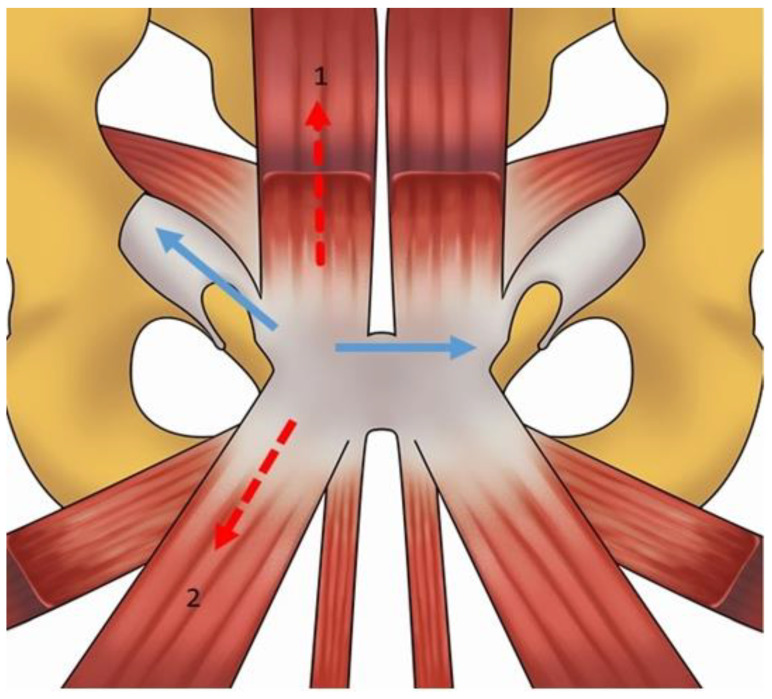
Vectors and shearing forces to which the prepubic aponeurotic complex is subjected. The red dotted arrows represent the vector forces, while the blue arrows represent the shearing forces. Specifically, the vector forces are represented by the force generated by the rectus abdominis muscle (vector facing up) and by the adductor muscles (vector facing down). During rotation and extension of the pelvis, the vector facing up creates the postero-superior tension, whilst the vector facing down creates the infero-anterior tension. Shearing forces are the result of applying a tangential force to a surface while the base remains stationary and are equal to the tangential component of the force over the contact area. Shearing forces tend to cause an opposite but parallel sliding motion of the planes of the prepubic aponeurotic complex. Legends: (1) rectus abdominis; (2) adductor longus.

The pelvis is composed of several bone structures including the ilium, ischium, pubis, sacrum and coccyx. These bones meet anteriorly at the midline to form the pubic symphysis. The pubic symphysis is a fibrous joint that distributes shear forces during ambulation. The joint is able to resist tensile, shearing and compressive forces with limited mobility. Indeed, in physiological conditions, it shows a maximum shift equal to 2 mm and a maximal rotation of 1° [16]. The most recent anatomical references classify the pubic symphysis as a secondary cartilaginous joint [17,18] or a fibrocartilaginous joint [19]. This type of classification has a stronger anatomical connotation than the older functional classification, in which the terms amphiarthrodial [20] or diarthrodial/amphiarthrodial were adopted to describe this joint [21]. Unfortunately, even today, several anatomical aspects of the pubic symphysis are not fully understood. Indeed, it is important to underline that the last anatomical study on the pubic symphysis was published in 1986 [22]. Therefore, this lack of recent anatomical studies represents an important limit to fully understanding the etiopathogenesis of certain clinical situations responsible for the onset of GPS. An important anatomical structure of the pelvis is the prepubic aponeurotic complex (PPAC), a schematic view of which is shown in Figure 1. The PPAC is formed by the interconnection between the tendons of the adductor longus, adductor brevis, gracilis and pectineus muscles; the aponeurosis of rectus abdominis, pyramidalis and external oblique muscles; the articular disc; the anterior pubic periosteum; and by the superior (SPLs), inferior (IPLs) and anterior pubic ligaments (APLs), while the posterior pubic ligament (PPL) is not part of the PPAC [23,24]. The SPL, IPL and APL are all in connection with the articular disc [16,25]. The PPAC new anatomical concept replaces the age-old accepted concept of the fusion of the rectus abdominis with the adductor longus via the aponeurotic plate [23,24,25,26]. Many anatomical studies focus on the APL due to its connection with the surrounding muscles as it brings together the inguinal ligament and the adductor longus and rectus abdominis muscles [16,23,24,25,26]. Furthermore, the adductor brevis muscle originates from both the APL and the IPL [25]. Therefore, from a biomechanical point of view, the APL is the anchorage point for both the superficial aponeurotic layers and the deep musculotendinous layers of the symphyseal region and may thus be considered the fulcrum of the PPAC [23,24]. In contrast, the IPL is considered to be the main stabilizer of the symphysis because of its thickness, its muscle connections and the different orientation of its fibers compared with the APL [16,26]. Again, from a biomechanical point of view, the symphysis is stabilized horizontally by the APL and vertically by the IPL and the SPL together, as reported by the morphometric data recorded by Pieroh et al. [25]. It is important to note that the APL is not as thick as either the SPL or the IPL [25] and these data could explain the greater force needed to induce a vertical displacement compared with a horizontal displacement of the symphysis [27]. As far as the elastic properties of the symphyseal ligaments are concerned, the only ligament with the possible presence of elastic fibers is the SPL [16]. Consequently, from a biomechanical point of view, the PPAC is an anatomical structure that presents an intrinsic stiffness [24] and, as it is subjected to important mechanical stress forces during athletic movements involving pelvic torsional movements, it also represents an area of biomechanical weakness [23,24]. Be that as it may, to date and to our knowledge, there are no reliable data on the biomechanical failure points of the symphyseal ligaments. Indeed, in certain pathologies, such as femoroacetabular impingement (FAI), the reduced motion of the hip joints is compensated by hypermobilization of the symphysis [28,29,30]. For this reason, FAI involves a high stress level for the APL, especially in the transverse plane [25,28], and it is probably of no coincidence that FAI frequently appears in the etiopathogenesis of GPS [2,13,29]. In addition, it must be remembered that the PPAC is subjected to both upward tensile forces from the rectus abdominis muscles and downward forces from the adductor muscles [24]. This pair of forces, in turn, generates weighty shearing forces [24,30] that can cause anatomical damage to the PPAC [13,24] and possibly trigger the onset of GPS. A schematic view of the forces to which the symphysis is subjected is shown in Figure 2.

**Figure 3:**
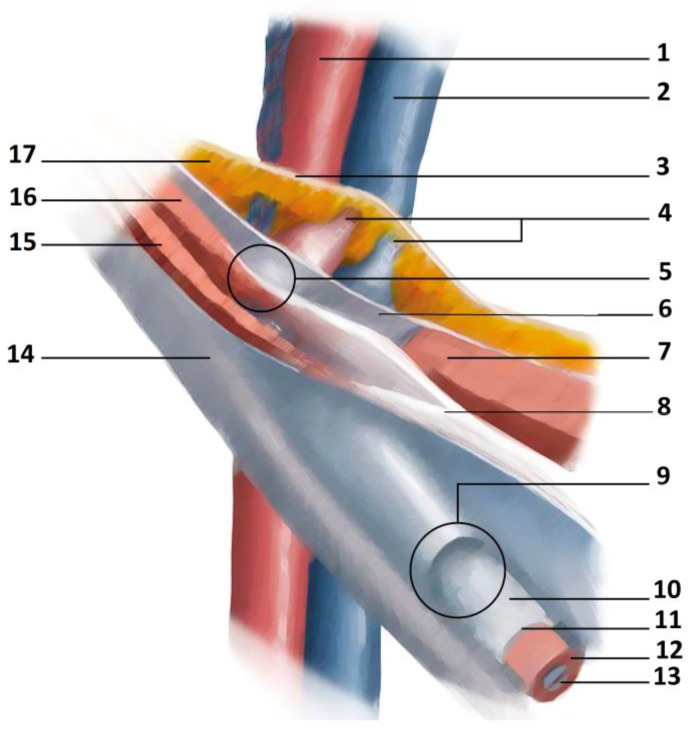
Schematic view of the inguinal canal. Legends: (1) external iliac artery; (2) external iliac vein; (3) parietal peritoneum; (4) inferior epigastric vessels; (5) internal inguinal ring; (6) transversalis fascia; (7) rectus abdominis muscle; (8) conjoint tendon; (9) external inguinal ring; (10) spermatic cord; (11) external spermatic fascia; (12) cremasteric muscle and fascia; (13) internal spermatic fascia; (14) external oblique muscle aponeurosis; (15) internal oblique muscle; (16) transversus oblique muscle; (17) extraperitoneal tissue.

Another important anatomical structure of the pelvis is the inguinal canal (IC). The IC is traversed by the spermatic cord in men and the round ligament in women. Four walls (anterior, inferior, superior and posterior) and two orifices or rings (one superficial or external and one deep) form the IC [31]. The aponeurosis of the external oblique muscle represents the main anatomical element of the anterior wall. The lower border of the internal oblique muscle and transversus muscle forms the upper wall of the inguinal canal. The posterior wall is formed by the transversalis fascia, which is strengthened laterally by the interfoveolar ligament of Hesselbach and medially by the ligament of Henle, the ligament of Colles and the conjoint tendon [32]. The superficial or external inguinal ring is delimited by the fibers of the aponeurosis of the external oblique muscle. These fibers originate from the anterior superior iliac spine [33]. The fibers leading up to the pubic tubercle form the inferior crus (infero-lateral pillar or external pillar), while the fibers leading up to the pubic symphysis form the superior crus (supero-medial pillar or internal pillar) [32]. The deep inguinal ring faces the abdominal cavity and is perpendicular to the middle part of the inguinal ligament. The deep inguinal ring is about 15–20 mm away from the inguinal ligament and about 50 mm from the pubic tubercle [31,33]. A schematic view of the inguinal canal is shown in Figure 3.

## 3. MRI Techniques

**Figure 4:**
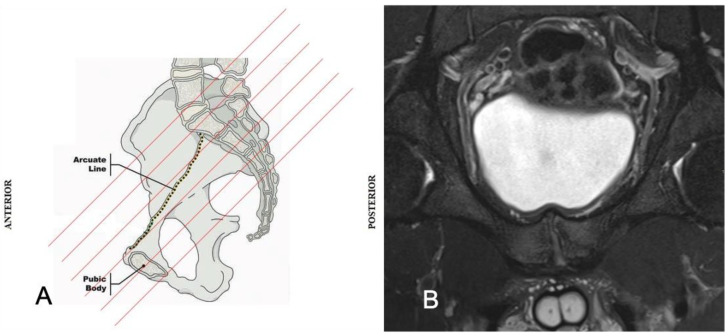
The oblique axial section is a sequence oriented in the sagittal plane parallel to the pelvic arcuate line along the medial surface of the hemipelvis (**A**). This sequence properly demonstrates the rectus abdominis muscle insertion and the proximal origin of the adductor muscles (**B**).

MRI and dynamic ultrasound (DUS) are currently the most effective imaging methods for diagnosing GPS. MRI is the best option for evaluating both osseous and soft tissue structures simultaneously. To evaluate GPS, the use of a scanner of at least 1.5 T and a non-contrast protocol is recommended. The recommended standard planes are coronal, sagittal and axial. However, axial and oblique coronal imaging are fundamental for PPAC assessment [13]). In particular, oblique axial imaging is indispensable for revealing the rectus abdominis muscle insertion and the proximal origin of the adductor muscles [14,34]. A graphical explanation of an oblique axial sequence is shown in Figure 4.

An MRI protocol for the evaluation of GPS is indicated in Table 1. Unfortunately, in most cases, a dynamic MRI protocol is not useful for diagnosing inguinal hernia or weakness of the posterior wall of the inguinal canal (the so-called sports hernia), as the inability of monitoring in real time the Valsalva maneuver that the patient must carry out during the MRI examination makes the number of false negatives unacceptably high [2,13,35,36]. It is important to note that MRI findings should always be interpreted considering the patient’s clinical history and physical examination together with other imaging studies, such as ultrasound or CT scans. In some cases, supplementary imaging studies may be necessary to confirm the diagnosis and to guide treatment. Overall, MRI is a valuable tool in the diagnosis and management of GPS because it allows for detailed evaluation of the pelvic structures and it facilitates accurate diagnosis and treatment planning.

## 4. GPS MRI Assessment

In the following sections, we describe some of the main clinical conditions causing GPS that can be investigated with MRI. However, it is important to remember that GPS can be caused by 67 different clinical conditions that can be divided into 12 nosological categories according to the taxonomic classification proposed by the Groin Pain Syndrome Italian Consensus Conference update 2023 [13]. This taxonomic classification is shown in Table 2. Consequently, MRI assessment alone may not always be sufficient for formulating a definitive diagnosis, and a multidisciplinary approach, based on various diagnostic imaging techniques, is required. We are advocates for the important contribution that MRI can bring to this multidisciplinary approach.

### 4.1. Prepubic Aponeurotic Complex Injuries 

The prepubic aponeurotic complex (PPAC) forms a fibrous capsule that lines the anterior of the pubic symphysis. The PPAC represents an area of biomechanical weakness that endures considerable stress forces during athletic movements involving pelvic torsional movements and single-stance maneuvers [13,16,23]. The most frequent clinical situations involving PPAC injuries are represented by the following two different anatomical damages [2,13,24]:

- Injury of the PPAC afferent to the adductor longus tendon–rectus abdominis–pyramidalis aponeurotic plate complex [24,37].
- PPAC avulsion from the anterior pubic bone [24,38].

**Figure 5:**
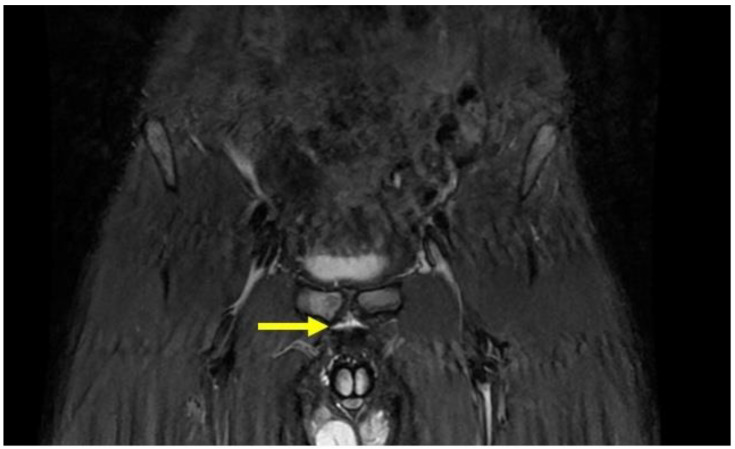
Coronal STIR-weighted image of 22-year old male athlete showing avulsion of the part of the pre-pubic aponeurotic complex afferent to the adductor longus tendon–rectus abdominis–pyramidalis aponeurotic plate complex. The hyperintensity signal extends bilaterally (arrow). Note the hyperintensity signal indicating the pre-pubic aponeurotic complex lesion that is not in continuity with the primary cleft sign.

**Figure 6:**
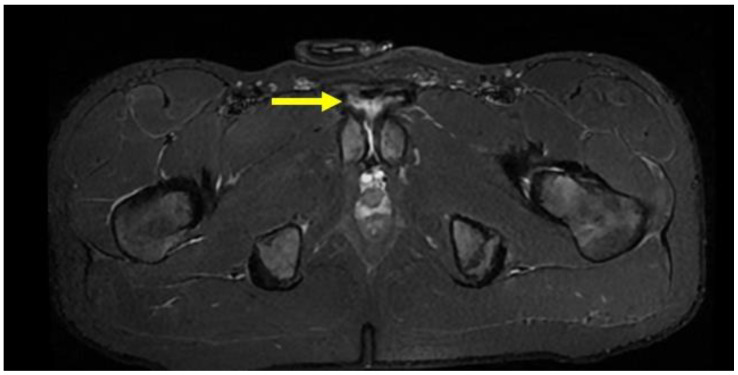
The same pre-pubic aponeurotic complex lesion (arrow) shown in Figure 4, observed in an axial STIR-weighted image.

**Figure 7:**
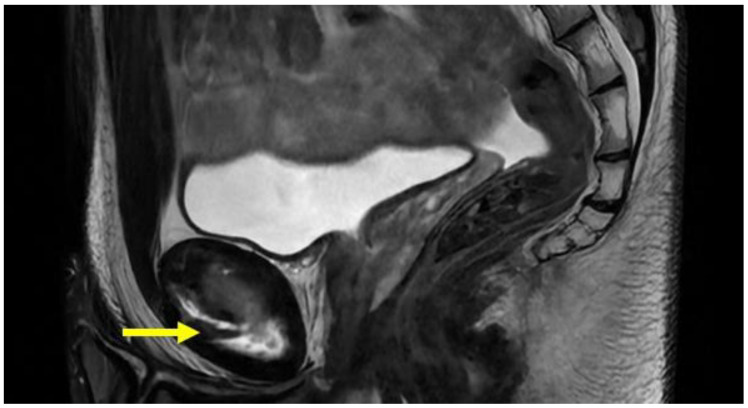
Sagittal T2 FSE-weighted image of 25-year old male athlete showing a pre-pubic aponeurotic complex avulsion from the anterior pubic bone (arrow).

PPAC injuries are visible upon MRI examination on all acquisition planes (axial, coronal and sagittal) in classic fluid-sensitive sequences (T2 and STIR) and in PD FS and intermediate FS sequences, but, as already mentioned, acquisitions of the oblique axial plane are particularly advisable. It is very important to make a distinction between the hyperintensity signal of a PPAC injury, observable in fluid-sensitive sequences due to a PPAC injury, and a hyperintensity signal concerning a secondary cleft sign. The secondary cleft sign is characterized by the presence of a high-intensity signal line extending laterally and downwards to the lower part of the symphysis. This line is always in communication with the symphysis joint space and it is mostly unilateral. Indeed, bilateral cleft signs are very rare [2,39,40]. In contrast, the hyperintensity signal of a PPAC injury is not in continuity with the symphysis joint space but originates at the midline of the PPAC and propagates either unilaterally or bilaterally and, in this latter instance, it will do so asymmetrically in most cases [41,42]. Furthermore, the anatomical location of a secondary cleft sign is different to that of a PPAC lesion: indeed, the secondary cleft sign is located inferiorly to the symphysis and inferiorly and posteriorly to the adductor longus pubic insertion [15,39,43]. A secondary cleft arises from a chronic maceration of the central fibrocartilaginous disc due to an excessive or abnormal mechanical stress and, if left untreated, with time, it may merge with the primary cleft sign (the physiological small central cavity of the articular disc) to varying degrees. For these reasons, the secondary cleft sign is considered a non-specific radiological sign that can be associated with different clinical situations such as an acute adductor longus tendon injury, a chronic tendinopathy or a dysfunction of the adductor longus, gracilis or conjoined tendons [15,43]. On the contrary, a hyperintensity signal due to a PPAC injury indicates a very specific lesion from an anatomical point of view, which is therefore very different from the secondary cleft sign. In the case of avulsion of the part of the PPAC afferent to the adductor longus tendon–rectus abdominis–pyramidalis aponeurotic plate complex, the signal is present unilaterally or bilaterally and is visible both in coronal and axial planes (Figure 5, Figure 6 and Figure 7). In the case of PPAC detachment from the pubic bone, a visible breach midline of the PPAC (Figure 7) is evident in the sagittal MRI sequences acquired. Furthermore, it is important to remember that the radiological signs of severe osteopathy are often part of the radiological presentation of a PPAC lesion.

### 4.2. Pubic Osteopathy

**Figure 8:**
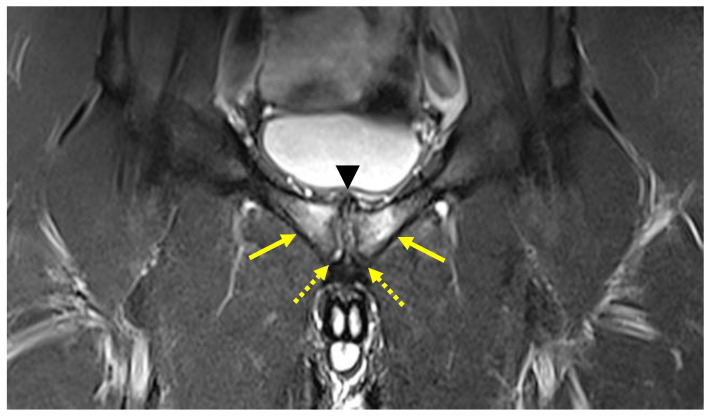
MRI in coronal STIR view showing central symphyseal disc protrusion (arrowhead), bilateral bone marrow oedema (arrows) and irregularities of the symphysis (dotted arrows). These radiological signs indicate severe pubic osteopathy.

In a GPS clinical situation, a cam-FAI often causes a reduction in normal hip intra-rotation. This ROM limitation is due to the impingement between the ball-shaped head of the femur and the articular rim [44]. This condition may cause an increase in the stiffness of the hip joint capsule [45], which is often compensated for by exaggerated mobilization of the symphyseal joint. In turn, this abnormal mobilization of the symphyseal joint may cause the onset of inguinal pathologies and pubic osteopathy and adductor tendinopathy [2,29,45,46]. As established by the Italian Consensus Conference on FAI Syndrome in Athletes Cotignola Agreement [47], the term pubic osteopathy is preferable to that of osteitis pubis as it is a chronic condition and not an inflammatory condition. It is possible to formulate the diagnosis of pubic osteopathy (Figure 8) when, in addition to the clinical condition, at least three of the following five radiological signs are present:

- Bone marrow oedema of the pubic branches;
- Signs of bone reabsorption and sclerosis of the pubic branches;
- Symphysis irregularity and/or signs of bone erosion;
- Subchondral cysts and/or osteophyte formations;
- Central disc protrusion.

Finally, it is important to remember that the radiological signs of severe osteopathy are frequently associated with cam-FAI [40] and PPAC injury [42], as already mentioned.

### 4.3. Adductor Muscle Injuries

**Figure 9:**
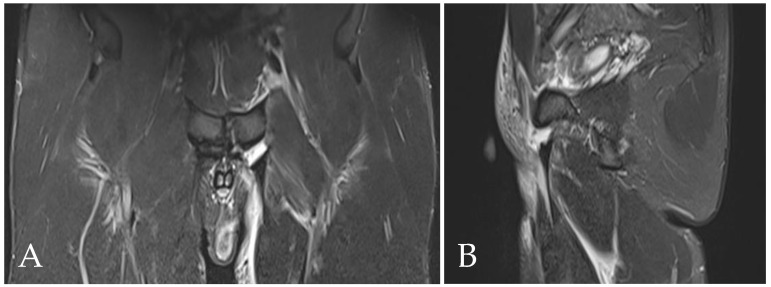
Coronal STIR (**A**) and sagittal STIR (**B**) showing a left adductor longus tendon pubic avulsion.

Adductor injuries are one of the most important causes of acute GPS [48]. The muscle most exposed to injury is the adductor longus (90% of cases) [48]. The anatomical location of adductor longus injuries is 25% proximal (of which 75% are avulsion injuries), 31% at the proximal muscle–tendon junction (mainly grade I and II), 37% at the distal tendons (predominantly grade I and II) and, for the remaining 7%, the location is intramuscular at the middle third (also predominantly grade I and II) (Figure 9) [49]. It is important to note that over 70% of adductor longus avulsions are PPAC injuries that are often overlooked [23,24]. The adductor longus shows two typical areas of injury: the first, antero-medial related to the proximal tendon; the second, postero-lateral related to the distal tendon [48]. Upon MRI examination, the sequences most suitable for studying adductor injuries are oblique axial (PD FS and T2 FS) and coronal STIR [50]. Classification of the degree of lesion must be calculated based on the relationship between its volume and the volume of the muscle [51,52].

### 4.4. Adductor Tendinopathy

As with injuries, the adductor muscle more prone to tendinopathy is the adductor longus [40]. Upon MRI examination, the sequences most suitable for the study of the adductor tendinopathies are as follows [15]:

- Oblique axial T1;
- Oblique axial PD FS; T2 FS and T1;
- Coronal T1.

**Figure 10:**
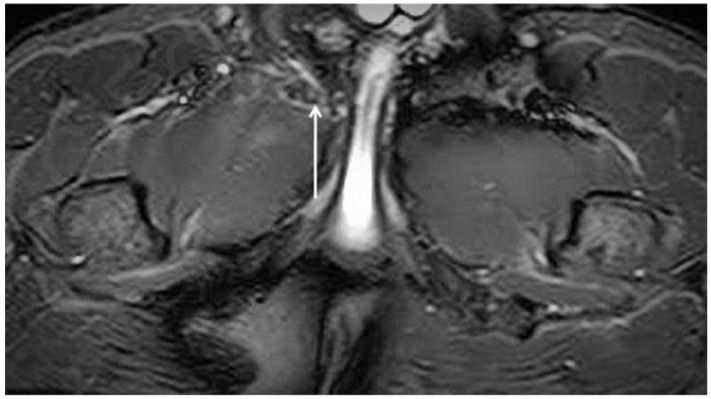
Axial oblique PD FS showing a right adductor longus tendinopathy (arrow).

Adductor longus tendinopathy (ALT), upon MRI examination, is represented by an increase in signal intensity at the adductor longus tendon and/or at its enthesis in the fluid-sensitive sequences (Figure 10). Tendon swelling and/or changes in enthesis morphology are also usually present. On the contrary, in normal physiological conditions, the tendon appears in all sequences hypointense, subtle and well defined, while in oblique axial sequences the tendon appears symmetric and triangular in shape, with the base facing the anterior margin of the pubic bone. On the contrary, in cases of ALT, the tendon may show a convex shape and increased signal intensity [14]. Unfortunately, no radiological grading scale is currently able to evaluate the severity of tendinopathy of the adductor longus [53]. However, it must be pointed out that, in some cases, ALT may reflect a functional adaptation in response to a functional overload incurred during strenuous sporting activities [43,54,55], and this functional adaptation may remain at a subclinical level or clinically manifest itself as a full-blown tendinopathy [40]. Finally, it is interesting to note that, in a clinical GPS situation, ALT is the most important radiological sign correlated to direct inguinal hernia and/or weakness of the inguinal posterior wall (i.e., the so-called sports hernia) with an odds ratio (OR) equal to 3.88 (OR 3.88; 1.27 to 11.54; 95% CI) [15], where OR measures the correlation between risk factors and outcome. OR represents the probability that an outcome will occur given a particular risk exposure compared with the probability of the outcome occurring in the absence of exposure to risk.

Obviously, this does not exclude the possibility of MRI detecting ALT without the presence of inguinal pathologies.

### 4.5. Rectus Abdominis Injuries

Isolated rectus abdominis muscle injury is not a common type of injury. These types of injuries are usually classified as rectus abdominis/adductor aponeurosis injuries. In MRI, the key indicator is, as in the case of any indirect muscle injury, an increased intramuscular signal on fluid-sensitive sequences. The MRI sequences most suitable for studying RAT are sagittal STIR and axial oblique PD FS [15]. Also, in this case, the classification of the degree of lesion must be calculated based on the relationship between its volume and the volume of the muscle. It is very important to remember that, as in the case of avulsions of the adductor longus, avulsions of the rectus abdominis may also cause a lesion of the PPAC [24].

### 4.6. Rectus Abdominis Tendinopathy

**Figure 11:**
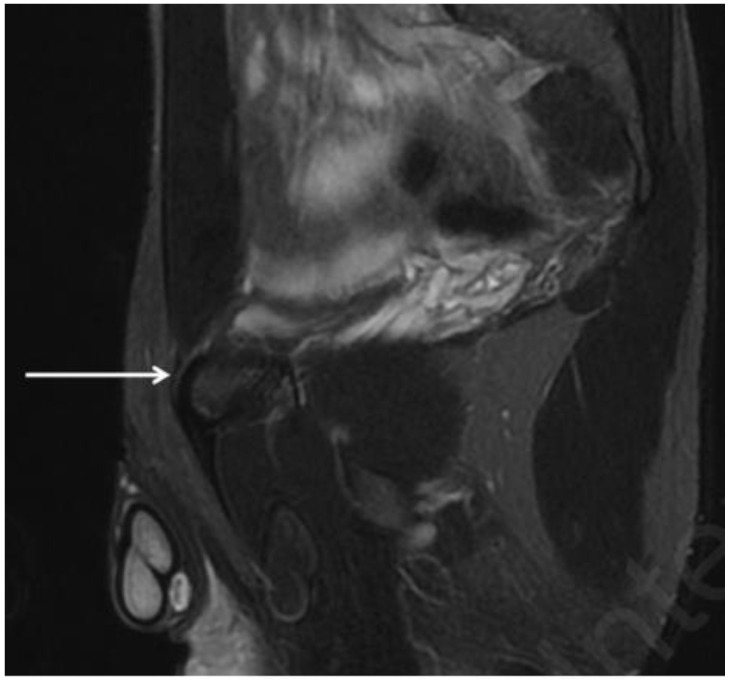
Sagittal STIR image showing signs of rectus abdominis tendinopathy (arrow).

Rectus abdominis tendinopathy (RAT) upon MRI examination appears as an increase in signal intensity in the fluid-sensitive sequence at the rectus abdominis muscle–tendon junction and/or an increase in the rectus abdominis tendon volume (Figure 11) [4,56]. As in the case of rectus abdominis muscle injuries, the MRI sequences most suitable for studying RAT are sagittal STIR and oblique axial PD FS [15]. RAT is rarely described in the literature. This may be partially explained by the fact that both the adductor longus and the rectus abdominis have a common insertion on the pubic symphysis, therefore several RATs are classified as ALT [57].

### 4.7. Inguinal Hernia

In LSGPS, the presence of inguinal hernias is different in the male and female population [40,58]. In the male population, a weakness of the posterior wall of the inguinal canal is present in 42.7% of cases, direct hernia in 8% of cases and external oblique hernia in 2% of cases. In the male population, inguinal pathologies are therefore responsible for over 50% of LSGPS cases [40]. In the female population affected by LSGPS, a weakness of the posterior wall of the inguinal canal is present in 37% of cases, direct hernia in 5.5% of cases, external oblique hernia in 2.7% of cases and femoral hernia in 10.8% of cases. Thus, in the female population, inguinal pathologies are also responsible for over 50% of LSGPS cases but their typology is different compared with the male population [58]. The radiological examination of choice for the study of inguinal and femoral hernias and for the investigation of the weakness of the posterior wall of the inguinal canal is DUS [2,13]. As already mentioned, a dynamic MRI protocol is not useful for diagnosing inguinal and femoral hernias or for diagnosing weakness of the posterior wall of the inguinal canal mainly due to the impossibility of monitoring in real time the Valsalva maneuver that the patient must carry out during the examination, which, in turn, causes an unacceptable number of false negatives [2,13,35,36]. However, MRI can be useful for detecting obturator hernias (OHs) [59], which, although rarer than inguinal and femoral hernias, may be an unrecognized cause of GPS from functional overload and/or of LSGPS [59,60]. A weakening of the obturator membrane may cause an enlargement of the obturator canal; consequently, the hernial contents may pass either anteromedially to the neurovascular bundle or by nudging the neurovascular bundle aside [59]. The obturator canal may be larger in men; for this reason, OHs show a 6:1 predominance female/male [61]. OHs are classified into the following three types [59]:

- Type I: the hernia contains only preperitoneal connective tissue and fat.
- Type II: the hernia progresses in the obturator canal and invaginates the peritoneal sac.
- Type III: the hernia presents further herniation of pelvic or peritoneal viscera such as bowel, bladder or ovary.

**Figure 12:**
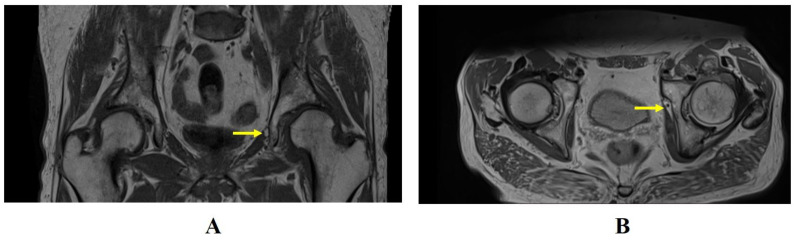
Coronal T1-weighted image (**A**) and axial PD TSE Dixon image (**B**). In both images, the right obturator canal is normal, while the left obturator canal demonstrates an abnormal volume of fat accompanying the obturator neurovascular bundle (arrow) as it passes between the obturator muscles. The images are compatible with a type I obturator hernia.

OH type I (Figure 12) is relatively rare and, for this reason, may present a diagnostic challenge for the radiologist. MRI sequences most suitable for the study of OH are the coronal and axial T1- or PD-weighted sequences. In MRI assessment, a protrusion of fat through the foramen between the pectineus and obturator externus muscles is a pathognomonic image for OH [35]. The fat may sometimes interpose between the adductor magnus and adductor brevis muscles along the course of the obturator nerve’s posterior division. The most important step of evaluation is the comparison of symmetry with the contralateral canal.

### 4.8. Hip Pathologies

**Figure 13:**
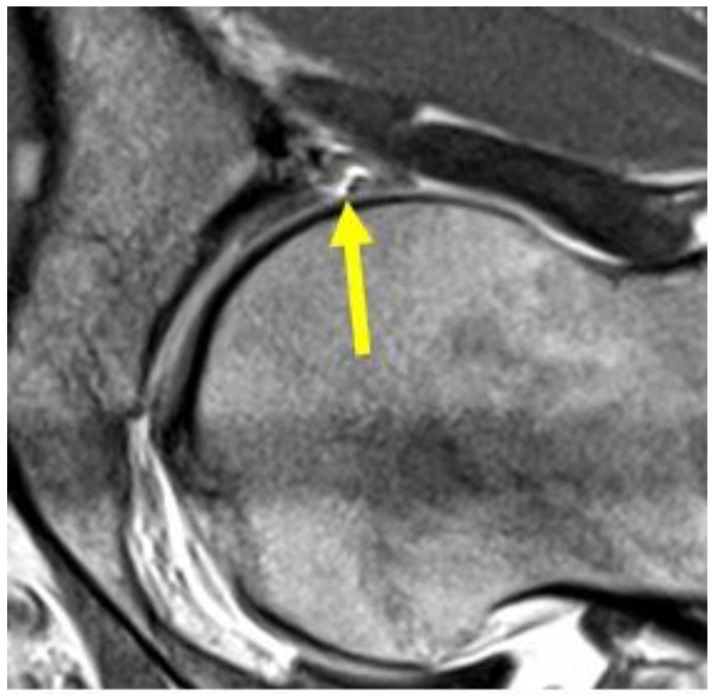
Acetabular labral lesion in MR arthrography coronal FSE PD sequence, where it is possible to observe the passage of contrast medium in the intralabral fissure (arrow).

MRI performed with 1.5 T or 3.0 T tomographs with the use of phased array surface coils is used for the panoramic study of the pelvis and the specific study of hip joint pathologies. The study protocol should include both wide (32–40 cm) FOV (field of view) and high resolution images acquired with narrower FOV (14–18 cm). The images acquired with narrower FOV are useful for evaluating the labral pathology and the chondral surface of the femoral heads. In this case, the acquisition sequences to use are fast spin echo (FSE) proton density (PD) in the axial, coronal and sagittal planes, with contiguous thin layer sections (3–4 mm) or with minimum interslice gap. Oblique axial images parallel to the femoral neck are used to identify any associated femoro-acetabular impingement [43]. MRI arthrography examination is the gold standard for chondral and labral damage assessment [62]. In MRI arthrography, labral injuries are confirmed by the contrast medium extending into the labral defect (Figure 13). To locate a labrum lesion, the clock face method is used with 3 o’clock anteriorly and 12 o’clock superiorly, regardless of the side of the hip [63]. The greatest number of acetabular labral lesions (84%) are located antero-superiorly, 16% occur postero-superiorly, while antero-inferior and postero-inferior lesions are rare [64]. The recommended protocol for MRI arthrography is as follows [65,66,67]:

- Coronal STIR (FOV 30–40 cm);
- Coronal PD or intermediate FS (FOV 16 cm),
- Sagittal or intermediate FS (FOV 16 cm),
- Radiant T1 or T1 FS.

### 4.9. Stress Fractures

**Figure 14:**
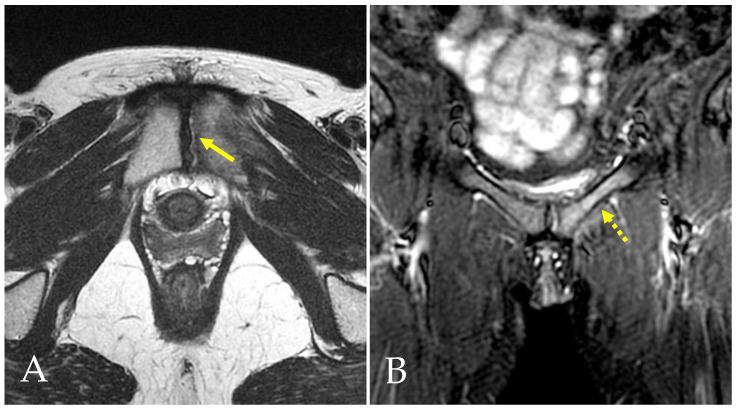
Axial T2 (**A**) and coronal STIR (**B**) MR images showing stress fracture as a linear subchondral hyperintensity (arrow). Coronal STIR image also shows diffuse BME of left pubic ramus (dotted arrow).

The most common locations for bone stress fractures in the pelvis are the medial femoral neck and the pubic rami. MRI examination reveals a signal hyperintensity in the fluid-sensitive sequences and a signal hypointensity in T1 sequences, which highlights the fracture line running perpendicular to the bone trabeculae [2]. The recommended sequences for stress fracture study are T1, T2 and STIR in coronal, sagittal and axial view [2,68] (Figure 14).

### 4.10. Symphyseal Apophysitis

**Figure 15:**
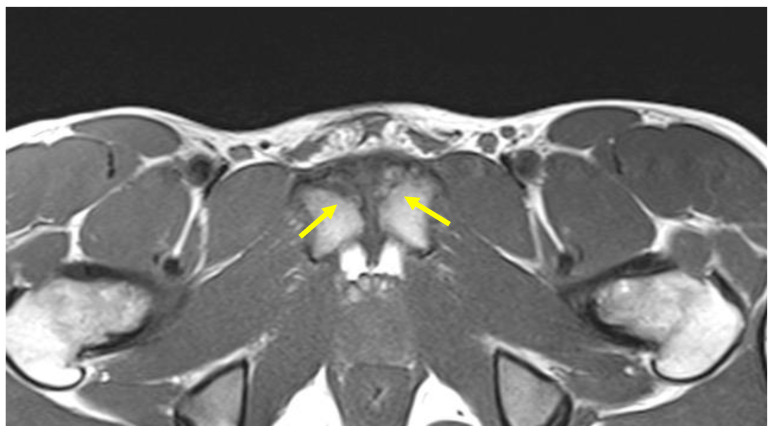
T1-weighted axial MR image of an eighteen-year-old patient showing two normal endochondral ossification nuclei (arrow).

Secondary ossification nuclei along the anteromedial aspect of the pubic bone appear during adolescence and reach complete ossification between the 20th and 25th years of life [69]. The presence of these active ossification nuclei could make the pubic bone particularly sensitive to the mechanical stress that is typical of some sports. It is important to remember that the presence of a growth plate separating the secondary ossification center from the pubic bone may be the cause of GPS due to apophysitis in the young athlete. This is a situation similar to the so-called little league shoulder or little league elbow, namely a pathology essentially due to a tension-overload mechanism of the growth plate [70]. It is interesting to note that some authors have defined this particular clinical situation with the term youth soccer groin [55,70,71]. The MRI sequence for the study of secondary ossification nuclei is coronal T1. The presence of active secondary ossification nuclei is confirmed by the signal hypointensity corresponding to the antero-medial ossification nucleus [2] (Figure 15).

### 4.11. Bone Marrow Edema

**Figure 16:**
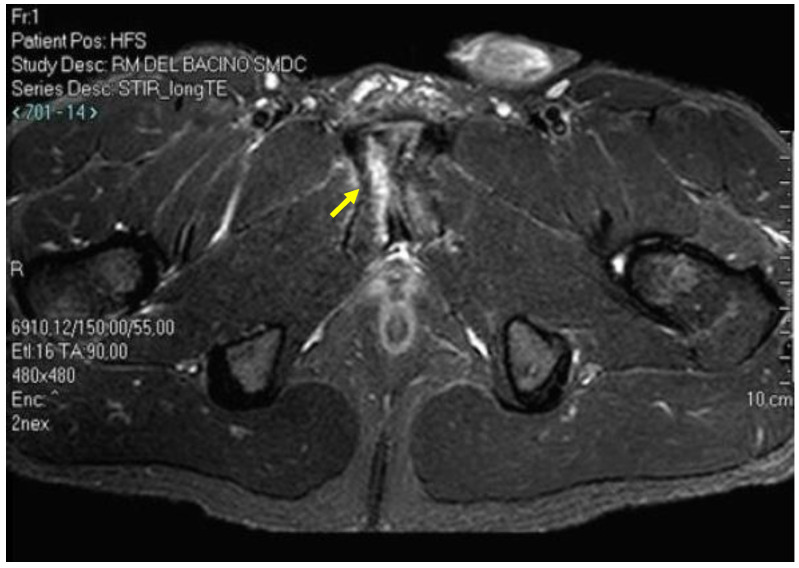
Axial STIR MRI showing a bone marrow oedema extending over the entire surface in an antero-posterior direction (arrow) of the right pubic ramus. Based on its extension, the bone marrow oedema is classified as Grade 3.

Bone marrow oedema (BMO) is the identification of an intra-osseous signal hyperintensity in correspondence with the margin of the pubic ramus in the fluid-sensitive sequences. In T1 sequences, the same areas showing signal hyperintensity in fluid-sensitive sequences must show signal hypointensity [72]. The sequences suggested for BMO study are as follows: coronal T1; coronal T2 FS; axial oblique T2 FS; and oblique axial PD FS [2,72]. The presence of BMO is found in approximately 80–90% of athletes affected by GPS [24,40,73]. However, it is not yet completely clear whether BMO is a simple marker of bone stress injury or whether it represents a primary source of pain in patients affected by GPS [71]. Indeed, a symphyseal BMO could, in an athlete, represent a normal sign of bone remodeling in answer to a high rate of mechanical stress [74]. It is important to remember that a fatty infiltration would represent a subsequent stage of aggravation compared with the simple presence of BMO [53,54]. BMO is classified into three grades based on its extension on the axial plane and it is measured in the oblique axial sequences PD FS or T2 FS along the long axis of the superior or inferior pubic ramus (Figure 16). The BMO grade is determined as follows: Grade 1: BMO ≤ 1 cm; Grade 2: BMO ≥ 1 cm and ≤2 cm; Grade 3: BMO ≥ 2 cm. Finally, it is important to remember that the presence of BMO is part of the diagnostic criteria of pubic osteopathy (see Section 4.2) [47].

## 5. Discussion

GPS affects both professional and amateur athletes. Indeed, it is an increasingly frequent problem in many sports involving cutting maneuvers, changes in direction and kicking, such as soccer, football, ice hockey, handball, tennis and rugby [2]. The etiology of GPS includes 12 nosological categories and 67 pathologies [13]. Furthermore, it is important to remember that LSGPS often represents a genuine diagnostic challenge for the clinician due to both the large number of clinical conditions that can cause GPS and the anatomical complexity of the pelvis [50,75]. Thus, the intrinsic complexity of GPS requires a multidisciplinary approach for a successful diagnosis [2] and there is no single imaging assessment that can be considered exhaustive in reaching a definitive diagnosis of GPS. Conventional radiology, DUS, MRI and CT are all capable of providing complementary information that is essential for diagnostic purposes. MRI, in particular, proves to be an imaging assessment of fundamental importance for a large number of pathologies (of which the main ones have been briefly described in this review) as it can be used both for confirming clinical diagnoses and for differential diagnoses [2,13,14]. Unfortunately, there is a lack of detailed descriptions of GPS MRI examination in the literature. Table 3 provides the main radiographic findings that the examiner should look for in a GPS condition (GPS of traumatic origin, GPS from functional overload and LSGPS), with the relevant definition and recommended sequences for their optimal visualization.

It is important to underline that, except for acute muscle–tendon injuries, some of the radiological findings listed in Table 3 may be found in symptomatic populations and in asymptomatic populations of athletes. In cases of asymptomaticity, these findings can be interpreted both as a functional adaptation to the biomechanics of the performance model and as a prodromal sign of a latent or paucisymptomatic pathology. In these situations, clinical analysis must refer to the patient’s clinical history, and diagnosis is challenging. In this regard, it is interesting to note that radiological signs of adductor longus tendinopathy are present in as many as 71% of asymptomatic subjects compared with 72% found in the population of symptomatic subjects [54]. It is obvious that we can raise some reasonable doubts about the fact that GPS is most frequently diagnosed as adductor tendinopathy [4,76,77]; indeed, in our recent study [40], we reported that adductor tendinopathy is responsible for only about 2% of cases of LSGPS. These data must undoubtedly cause us to reflect on how imprudent it is to stop at a simple diagnosis of this type without considering other possible pathological associations [24,40].

### Limitations of the Study

This study has several limitations. Due to the lack of space, only the musculoskeletal clinical conditions causing GPS are described, whereas the visceral or neurological clinical conditions causing GPS that can be investigated, respectively, with MRI and neuro-MRI are not considered. Furthermore, again for reasons of space, it is not possible to include a comparison between the MRI images and those derived from other diagnostic investigation methods (X-ray, computerized axial tomography, ultrasound).

## 6. Conclusions

GPS is undoubtedly a complex clinical situation that engages the clinician in a significant way. This complexity depends both on the anatomical intricacy of the pelvis and on the numerous clinical conditions that may cause GPS. For all these reasons, a multidisciplinary approach is an essential requirement for a successful diagnosis. In this context, imaging to support clinical reasoning, of which MRI represents an important aspect, can help the clinician overcome the diagnostic challenge that GPS imposes.

### Future Directions

MRI is an imaging method undergoing constant technical evolution. Indeed, MRI has made remarkable progress in the last decade [78]. Therefore, in the near future, the evolution of techniques such as the delayed gadolinium-enhanced MRI of cartilage (dGEMRIC) [79] and T2 mapping at 3T MRI may allow for an accurate study of cartilaginous lesions of the hip and labral lesions. These new and promising techniques could therefore replace MRI arthrography, limiting the invasiveness of examinations.

## Figures and Tables

**Table 1:** MRI non-contrast protocol recommended for GPS assessment.

Acquisition Plan	Sequences	Slice (Max)	FOV (Max)
Entire pelvis coronal	STIR	5 mm	32–40 cm
Coronal	T1	3 mm	14–18 cm
Axial	T2	3 mm	14–18 cm
Oblique axial	PD FS or Intermediate FS	3 mm	14–18 cm
Oblique coronal	PD FS or Intermediate FS	3 mm	14–18 cm

Legends: STIR—short time inversion recovery. PD-FS—proton density fat-saturated. Intermediate FS—intermediate fat-saturated. FOV—field-of-view.

**Table 2:** Taxonomic classification proposed by the Groin Pain Syndrome Italian Consensus Conference update 2023 [13], subdivided into 12 nosological categories, including 67 possible different clinical conditions.

**(1) Articular causes**
Acetabular labrum tear
Femoroacetabular impingement (FAI)
Hip osteoarthritis
Intra-articular loose bodies
Hip instability
Adhesive capsulitis
Legg–Calvé–Perthes disease and its outcomes
Dysplasia and its outcomes
Epiphysiolysis and its outcomes
Avascular necrosis of the femoral head
Sacroiliac joint disorders
Lumbar spine disorders
Synovitis
**(2) Extra-articular causes**
Anterior inferior iliac spine impingement
Hip antero-superior labral tear with avulsion of rectus femoris
Ischiofemoral impingement syndrome
**(3) Visceral causes**
Inguinal hernia
Other types of abdominal hernia
Intestinal diseases
**(4) Bone causes**
Fractures and their outcomes
Stress fractures
Avulsion fractures
Iliac crest contusion (hip pointers)
**(5) Musculotendinous causes**
Rectus abdominis injuries and/or tendinopathy
Adductors muscles injuries and/or tendinopathy
Rectus abdominis–adductor longus common aponeurosis injuries
Iliopsoas injuries and/or tendinopathy
Prepubic aponeurotic complex (PPAC) injuries
Other indirect muscle injuries and their outcomes
Direct muscle injuries
Iliopsoas impingement (I)
Snapping internal or external hip
Bursitis
Weakness of the inguinal canal posterior wall
**(6) Pubic symphysis-related causes**
Osteitis pubis
Symphysis instability
Symphysis degenerative arthropathy
**(7) Neurological causes**
Nerve entrapment syndrome
Anterior cutaneous nerve entrapment syndrome
**(8) Developmental causes**
Apophysitis
Growth plate at pubic level
**(9) Genitourinary disease-related causes (inflammatory and non-inflammatory)**
Prostatitis
Epididymitis
Corditis
Orchitis
Varicocele
Hydrocele
Urethritis
Other infections of the urinary tract
Cystitis
Ovarian cysts
Endometriosis
Ectopic pregnancy
Round ligament entrapment
Testicular/ovarian torsion
Ureteral lithiasis
**(10) Neoplastic causes**
Testicular carcinoma
Osteoid osteoma
Other carcinomas
**(11) Infectious causes**
Osteomyelitis
Septic arthritis
**(12) Systemic causes**
Inguinal lymphadenopathy
Rheumatic diseases

**Table 3:** MRI findings of clinical relevance in GPS.

Pathologies	RM Sequences	RM Findings
PPAC injuries	T2, STIR, PD FS and intermediate FS sequences in axial, coronal and sagittal plans	Signal hyperintensity in fluid-sensitive sequences.
Adductor muscle injuries	Axial oblique PD FS and T2 FS. Coronal STIR	Signal hyperintensity in fluid-sensitive sequences.
Adductor tendinopathy	Axial oblique T1; axial oblique PD FS; T2 FS and T1; coronal T1	Increased signal intensity at the tendon level and/or at its enthesis level in the fluid-sensitive sequences. Tendon swelling and/or changes in enthesis morphology.
Rectus abdominis injuries	Sagittal STIR and axial oblique PD FS	Signal hyperintensity in fluid-sensitive sequences.
Rectus abdominis tendinopathy	Sagittal STIR and axial oblique PD FS	Increased signal intensity in the fluid-sensitive sequence at rectus abdominis muscle–tendon junction level and/or an increased rectus abdominis tendon volume.
Obturator hernia	Coronal and axial T1- and PD-weighted sequences	Protrusion of fat through the foramen between the pectineus and obturator externus muscles. Very important evaluation of the comparison for symmetry with the contralateral canal.
Acetabular labrum lesion	MRI arthrography: coronal STIR (FOV 30–40 cm); coronal PD or intermediate FS (FOV 16 cm); sagittal or intermediate FS (FOV 16 cm); radiant T1 or T1 FS.	Spreading of the contrast medium into the labral defect.
Stress fractures	T1, T2 and STIR in coronal, sagittal and axial view	Signal hyperintensity in the fluid-sensitive sequences and signal hypointensity in T1 sequences.
Symphyseal apophysitis	Coronal T1; axial T1	Signal hypointensity corresponding to the anteromedial ossification nucleus.
Bone marrow oedema	Coronal T1; coronal T2 FS; axial oblique T2 FS; axial oblique PD FS	Signal hyperintensity in the fluid-sensitive sequences. Signal hypointensity in T1 sequences. Grade 1: BMO ≤ 1 cm; Grade 2: BMO ≥ 1 cm and ≤2 cm; Grade 3: BMO ≥ 2 cm.
Subchondral cyst	Coronal STIR. Axial oblique T2	Presence of subchondral cyst (hyperintense subchondral cystic element in fluid-sensitive sequences).
Central disc protrusion	Coronal T1. Axial oblique T1	Protrusion of the central symphyseal fibrous disc. In coronal images, the central disc protrudes cranially with respect to the margins of the symphyseal joint. In oblique axial sequences, it protrudes posteriorly.
Secondary inferior cleft sign	Coronal STIR. Axial oblique PD FS	High signal intensity line extending laterally and inferiorly to the lower part of the symphysis, which appears to be in communication with the symphyseal joint space.
Secondary superior cleft sign	Coronal STIR. Axial oblique PD FS	High signal intensity line in fluid-sensitive sequences extending parallel to the inferior border of the superior pubic ramus shows connection with the symphyseal joint space.
Sclerosis of the symphysis	Coronal T1; axial oblique T1	Presence of bone sclerosis along the articular margins of the symphysis. The sclerotic area appears as hypointense bone formation (increased thickness) along the articular margins of the symphysis.
Fatty infiltration	Coronal T1; coronal STIR; axial oblique T2 FS; axial oblique PD FS	Areas of high signal intensity at the level of the symphysis in T1-weighted sequences and areas of low signal intensity in fat-saturated sequences

Notes: An MRI finding is considered present (i.e., positive) only if visible on at least two different image planes. When two differently weighted sequences acquired on the same plane are compared, attention must be paid to using the same table position. In case of doubt concerning the presence or absence of an MRI finding, the latter is to be considered absent.

## Data Availability

No new data were created in this study. The data relating to the images (MRI) are not available for patient privacy reasons.

## References

[1] Bisciotti G.N., Volpi P., Zini R., Auci A., Aprato A., Belli A., Bellistri G., Benelli P., Bona S., Bonaiuti D. (2016). Groin Pain Syndrome Italian Consensus Conference on terminology, clinical evaluation and imaging assessment in groin pain in athlete. BMJ Open Sport Exerc. Med..

[2] Mosler A.B., Weir A., Eirale C., Farooq A., Thorborg K., Whiteley R.J., Hölmich P., Crossley K.M. (2018). Epidemiology of time loss groin injuries in a men’s professional football league: A 2-year prospective study of 17 clubs and 606 players. Br. J. Sports Med..

[3] Hölmich P. (2007). Long-standing groin pain in sportspeople falls into three primary patterns, a “clinical entity” approach: A prospective study of 207 patients. Br. J. Sports Med..

[4] Waldén M., Hägglund M., Ekstrand J. (2015). The epidemiology of groin injury in senior football: A systematic review of prospective studies. Br. J. Sports Med..

[5] Junge A., Dvorak J. (2004). Soccer injuries: A review on incidence and prevention. Sports Med..

[6] Dvorak J., Junge A., Derman W., Schwellnus M. (2011). Injuries and illnesses of football players during the 2010 FIFA World Cup. Br. J. Sports Med..

[7] Bjørneboe J., Bahr R., Andersen T.E. (2014). Gradual increase in the risk of match injury in Norwegian male professional football: A 6-year prospective study. Scand. J. Med. Sci. Sports.

[8] Noya Salces J., Gómez-Carmona P.M., Gracia-Marco L., Moliner-Urdiales D., Sillero-Quintana M. (2014). Epidemiology of injuries in First Division Spanish football. J. Sports Sci..

[9] Harøy J., Andersen T.E., Bahr R. (2017). Groin Problems in Male Soccer Players Are More Common Than Previously Reported: Response. Am. J. Sports Med..

[10] Bahr R. (2009). No injuries, but plenty of pain? On the methodology for recording overuse symptoms in sports. Br. J. Sports Med..

[11] Werner J., Hägglund M., Ekstrand J., Waldén M. (2019). Hip and groin time-loss injuries decreased slightly but injury burden remained constant in men’s professional football: The 15-year prospective UEFA Elite Club Injury Study. Br. J. Sports Med..

[12] Bisciotti G.N., Zini R., Aluigi M., Aprato A., Auci A., Bellinzona E., Benelli P., Bigoni M., Bisciotti A., Bisciotti A. (2023). Groin Pain Syndrome Italian Consensus Conference update 2023. J. Sports Med. Phys. Fit..

[13] Omar I.M., Zoga A.C., Kavanagh E.C., Koulouris G., Bergin D., Gopez A.G., Morrison W.B., Meyers W.C. (2008). Athletic pubalgia and “sports hernia”: Optimal MR imaging technique and findings. Radiographics.

[14] Bisciotti G., Auci A., Cena E., Corsini A., Bisciotti A., Eirale C., Parra F., Gassaghi G., Di Marzo F., Vuckvovic Z. (2018). Potential MRI findings associated with inguinal hernia and inguinal canal posterior wall weakness in athletes. MLTJ.

[15] Becker I., Woodley S.J., Stringer M.D. (2010). The adult human pubic symphysis: A systematic review. J. Anat..

[16] McMinn R.M. (1994). Last’s Anatomy. Regional and Applied.

[17] Standring S. (2008). Gray’s Anatomy: The Anatomical Basis of Clinical Practice.

[18] Rosse C., Gaddum-Rosse P. (1997). Hollinshead’s Textbook of Anatomy.

[19] Gray H. (1858). Anatomy: Descriptive and Surgical.

[20] Testut J., Latarjet A. (1928). Traite d’Anatomie Humaine.

[21] Gamble J.G., Simmons S.C., Freedman M. (1986). The symphysis pubis. Anatomic and pathologic considerations. Clin. Orthop. Relat. Res..

[22] Schilders E., Mitchell A.W.M., Johnson R., Dimitrakopoulou A., Kartsonaki C., Lee J.C. (2021). Proximal adductor avulsions are rarely isolated but usually involve injury to the PLAC and pectineus: Descriptive MRI findings in 145 athletes. Knee Surg. Sports Traumatol. Arthrosc..

[23] Bisciotti A., Bisciotti G.N., Eirale C., Bisciotti A., Auci A., Bona S., Zini R. (2022). Prepubic aponeurotic complex injuries: A structured narrative review. J. Sports Med. Phys. Fit..

[24] Pieroh P., Li Z.L., Kawata S., Ogawa Y., Josten C., Steinke H., Dehghani F., Itoh M. (2021). The topography and morphometrics of the pubic ligaments. Ann. Anat..

[25] Mathieu T., Van Glabbeek F., Van Nassauw L., Van Den Plas K., Denteneer L., Stassijns G. (2022). New insights into the musculotendinous and ligamentous attachments at the pubic symphysis: A systematic review. Ann. Anat..

[26] Meissner A., Fell M., Wilk R., Boenick U., Rahmanzadeh R. (1996). Zur Biomechanik der Symphyse. Welche Kräfte führen zur Mobilität der Symphyse unter physiologischen Bedingungen? [Biomechanics of the pubic symphysis. Which forces lead to mobility of the symphysis in physiological conditions?]. Unfallchirurg.

[27] Birmingham P.M., Kelly B.T., Jacobs R., McGrady L., Wang M. (2012). The effect of dynamic femoroacetabular impingement on pubic symphysis motion: A cadaveric study. Am. J. Sports Med..

[28] Bisciotti G.N., Di Marzo F., Auci A., Parra F., Cassaghi G., Corsini A., Petrera M., Volpi P., Vuckovic Z., Panascì M. (2017). Cam morphology and inguinal pathologies: Is there a possible connection?. J. Orthop. Traumatol..

[29] Radin E.L., Sheldon R.S. (1979). Practical Biomechanics for the Orthopedic Surgeon.

[30] Tuma F., Lopez R.A., Varacallo M. (2023). Anatomy, Abdomen and Pelvis: Inguinal Region (Inguinal Canal). StatPearls [Internet].

[31] Lytle W.J. (1979). Inguinal anatomy. J. Anat..

[32] HerniaSurge Group (2018). International guidelines for groin hernia management. Hernia.

[33] Todeschini K., Daruge P., Bordalo-Rodrigues M., Pedrinelli A., Busetto A.M. (2019). Imaging Assessment of the Pubis in Soccer Players. Rev. Bras. Ortop..

[34] Aguirre D.A., Santosa A.C., Casola G., Sirlin C.B. (2005). Abdominal wall hernias: Imaging features, complications, and diagnostic pitfalls at multi-detector row CT. Radiographics.

[35] Garvey J.F., Read J.W., Turner A. (2010). Sportsman hernia: What can we do?. Hernia.

[36] Mercouris P. (2014). Sports hernia: A pictorial review. SA J. Radiol..

[37] Matsuda D.K., Matsuda N.A., Head R., Tivorsak T. (2017). Endoscopic Rectus Abdominis and Prepubic Aponeurosis Repairs for Treatment of Athletic Pubalgia. Arthrosc. Tech..

[38] Brennan D., O’connell M.J., Ryan M., Cunningham P., Taylor D., Cronin C., O’neill P., Eustace S. (2005). Secondary cleft sign as a marker of injury in athletes with groin pain: MR image appearance and interpretation. Radiology.

[39] Bisciotti G.N., Auci A., Bona S., Bisciotti A., Bisciotti A., Cassaghi G., Di Marzo F., Di Pietto F., Eirale C., Panascì M. (2021). A multidisciplinary assessment of 320 athletes with long-standing groin pain syndrome in keeping with the Italian consensus agreement: The high incidence and the multiple causes of inguinal and hip pathologies and pubic osteopathy. J. Sports Med. Phys. Fit..

[40] Khan W., Zoga A.C., Meyers W.C. (2013). Magnetic resonance imaging of athletic pubalgia and the sports hernia: Current understanding and practice. Magn. Reson. Imaging Clin. N. Am..

[41] Palisch A., Zoga A.C., Meyers W.C. (2013). Imaging of athletic pubalgia and core muscle injuries: Clinical and therapeutic correlations. Clin. Sports Med..

[42] Cunningham P.M., Brennan D., O‘Connell M., MacMahon P., O‘Neill P., Eustace S. (2007). Patterns of bone and soft-tissue injury at the symphysis pubis in soccer players: Observations at MRI. AJR Am. J. Roentgenol..

[43] Gerhardt M.B., Romero A.A., Silvers H.J., Harris D.J., Watanabe D., Mandelbaum B.R. (2012). The prevalence of radiographic hip abnormalities in elite soccer players. Am. J. Sports Med..

[44] Verrall G.M., Hamilton I.A., Slavotinek J.P., Oakeshott R.D., Spriggins A.J., Barnes P.G., Fon G.T. (2005). Hip joint range of motion reduction in sports-related chronic groin injury diagnosed as pubic bone stress injury. J. Sci. Med. Sport.

[45] Taylor R., Vuckovic Z., Mosler A., Agricola R., Otten R., Jacobsen P., Holmich P., Weir A. (2018). Multidisciplinary Assessment of 100 Athletes With Groin Pain Using the Doha Agreement: High Prevalence of Adductor-Related Groin Pain in Conjunction With Multiple Causes. Clin. J. Sport. Med..

[46] Zini R., Panascì M., Santori N., Potestio D., Di Pietto F., Bisciotti G.N. (2023). The Italian Consensus Conference on FAI Syndrome in Athletes (Cotignola Agreement). MLTJ.

[47] Serner A., Tol J.L., Jomaah N., Weir A., Whiteley R., Thorborg K., Robinson M., Hölmich P. (2015). Diagnosis of Acute Groin Injuries: A Prospective Study of 110 Athletes. Am. J. Sports Med..

[48] Serner A., Weir A., Tol J.L., Thorborg K., Roemer F., Guermazi A., Yamashiro E., Hölmich P. (2018). Characteristics of acute groin injuries in the adductor muscles: A detailed MRI study in athletes. Scand. J. Med. Sci. Sports.

[49] De Maeseneer M., Forsyth R., Provyn S., Milants A., Lenchik L., De Smet A., Marcelis S., Shahabpour M. (2019). MR imaging-anatomical-histological evaluation of the abdominal muscles, aponeurosis, and adductor tendon insertions on the pubic symphysis: A cadaver study. Eur. J. Radiol..

[50] Bisciotti G.N., Volpi P., Alberti G., Aprato A., Artina M., Auci A., Bait C., Belli A., Bellistri G., Bettinsoli P. (2019). Italian consensus statement (2020) on return to play after lower limb muscle injury in football (soccer). BMJ Open Sport. Exerc. Med..

[51] Coppola L., Canonico R., De Luca G., Bisciotti G.N., Rusconi G., Barillaro A., Di Pietto F. (2024). Magnetic resonance imaging predicts the days lost from training and competition: Evaluation of 56 indirect muscle injuries in professional football players. J. Sports Med. Phys. Fit..

[52] Branci S., Thorborg K., Nielsen M.B., Hölmich P. (2013). Radiological findings in symphyseal and adductor-related groin pain in athletes: A critical review of the literature. Br. J. Sports Med..

[53] Branci S., Thorborg K., Bech B.H., Boesen M., Nielsen M.B., Hölmich P. (2015). MRI findings in soccer players with long-standing adductor-related groin pain and asymptomatic controls. Br. J. Sports Med..

[54] Paajanen H., Hermunen H., Ristolainen L., Branci S. (2019). Long-standing groin pain in contact sports: A prospective case-control and MRI study. BMJ Open Sport. Exerc. Med..

[55] Werner J., Hägglund M., Waldén M., Ekstrand J. (2009). UEFA injury study: A prospective study of hip and groin injuries in professional football over seven consecutive seasons. Br. J. Sports Med..

[56] Bisciotti G.N., Chamari K., Cena E., Garcia G.R., Vuckovic Z., Bisciotti A., Bisciotti A., Zini R., Corsini A., Volpi P. (2021). The conservative treatment of longstanding adductor-related groin pain syndrome: A critical and systematic review. Biol. Sport..

[57] Bisciotti G.N., Auci A., Bona S., Bisciotti A., Bisciotti A., Cassaghi G., Di Marzo F., Di Pietto F., Eirale C., Panascì M. (2022). Long-standing groin pain syndrome in athletic women: A multidisciplinary assessment in keeping with the Italian Consensus Agreement. J. Sports Med. Phys. Fit..

[58] Droukas D.D., Zoland M.P., Klein D.A. (2019). Radiographic and surgical findings of type I obturator hernias in patients with refractory groin pain. Clin. Imaging.

[59] Mercado M., Diab J., Loi K. (2021). A delayed diagnosis of obturator hernia hoodwinked by previous laparoscopic inguinal hernia repair. J. Surg. Case Rep..

[60] Fitzgibbons R.J., Forse R.A. (2015). Clinical practice. Groin hernias in adults. N. Engl. J. Med..

[61] Hesper T., Neugroda C., Schleich C., Antoch G., Hosalkar H., Krauspe R., Zilkens C., Bittersohl B. (2018). T2*-Mapping of Acetabular Cartilage in Patients With Femoroacetabular Impingement at 3 Tesla: Comparative Analysis with Arthroscopic Findings. Cartilage.

[62] Blankenbaker D.G., De Smet A.A. (2010). Hip injuries in athletes. Radiol. Clin. N. Am..

[63] Sutter R., Zubler V., Hoffmann A., Mamisch-Saupe N., Dora C., Kalberer F., Zanetti M., Hodler J., Pfirrmann C.W. (2014). Hip MRI: How useful is intraarticular contrast material for evaluating surgically proven lesions of the labrum and articular cartilage?. AJR Am. J. Roentgenol..

[64] Llopis E., Fernandez E., Cerezal L. (2012). MR and CT arthrography of the hip. Semin. Musculoskelet. Radiol..

[65] Naraghi A., White L.M. (2015). MRI of Labral and Chondral Lesions of the Hip. AJR Am. J. Roentgenol..

[66] Weishuhn L.J., Seidman A. (2023). Hip Arthrogram. StatPearls [Internet].

[67] Bernstein E.M., Kelsey T.J., Cochran G.K., Deafenbaugh B.K., Kuhn K.M. (2022). Femoral Neck Stress Fractures: An Updated Review. J. Am. Acad. Orthop. Surg..

[68] Dimitrakopoulou A., Schilders E. (2016). Current concepts of inguinal-related and adductor-related groin pain. Hip Int..

[69] Kijowski R., Tuite M.J. (2010). Pediatric throwing injuries of the elbow. Semin. Musculoskelet. Radiol..

[70] Rubin A.D., Diduch D.R., Brunt L.M. (2014). Imaging in athletic groin pain. Sports Hernia and Athletic Pubalgia.

[71] Nakayama K., Utsunomiya H., Murata Y., Takada S., Tsukamoto M., Sakai A., Uchida S. (2022). Cleft Sign and Bone Marrow Edema of the Pubic Symphysis Are Associated With Sports and Bony Morphology in Patients with Femoroacetabular Impingement and Labral Tears. Orthop. J. Sports Med..

[72] Verrall G.M., Slavotinek J.P., Fon G.T. (2001). Incidence of pubic bone marrow oedema in Australian rules football players: Relation to groin pain. Br. J. Sports Med..

[73] Zoga A.C., Mullens F.E., Meyers W.C. (2010). The spectrum of MR imaging in athletic pubalgia. Radiol. Clin. N. Am..

[74] Riff A.J., Movassaghi K., Beck E.C., Neal W.H., Inoue N., Coleman S.H., Nho S.J. (2019). Surface Mapping of the Musculotendinous Attachments at the Pubic Symphysis in Cadaveric Specimens: Implications for the Treatment of Core Muscle Injury. Arthroscopy.

[75] Hölmich P., Uhrskou P., Ulnits L., Kanstrup I.L., Nielsen M.B., Bjerg A.M., Krogsgaard K. (1999). Effectiveness of active physical training as treatment for long-standing adductor-related groin pain in athletes: Randomised trial. Lancet.

[76] DeLang M.D., Garrison J.C., Hannon J.P., McGovern R.P., Sheedy P.J., Christoforetti J.J., Thorborg K. (2022). Midseason Screening for Groin Pain, Severity, and Disability in 101 Elite American Youth Soccer Players: A Cross-Sectional Study. Clin. J. Sport. Med..

[77] Bordalo M., Arnaiz J., Yamashiro E., Al-Naimi M.R. (2023). Imaging of Muscle Injuries: MR Imaging-Ultrasound Correlation. Magn. Reson. Imaging Clin. N. Am..

[78] Lee J.H., Houck D.A., Gruizinga B.A., Garabekyan T., Jesse M.K., Kraeutler M.J., Mei-Dan O. (2022). Correlation of Delayed Gadolinium-Enhanced MRI of Cartilage (dGEMRIC) Value With Hip Arthroscopy Intraoperative Findings and Midterm Periacetabular Osteotomy Outcomes. Orthop. J. Sports Med..

[79] Shoji T., Yamasaki T., Izumi S., Sawa M., Akiyama Y., Yasunaga Y., Adachi N. (2018). Evaluation of articular cartilage following rotational acetabular osteotomy for hip dysplasia using T2 mapping MRI. Skelet. Radiol..

